# Paternal alcohol exposure and dental-facial anomalies in offspring. Reply. 

**DOI:** 10.1172/JCI174216

**Published:** 2023-10-02

**Authors:** Kara N. Thomas, Destani D. Derrico, Michael C. Golding

**Affiliations:** Department of Veterinary Physiology and Pharmacology, School of Veterinary Medicine and Biomedical Sciences, Texas A&M University, College Station, Texas, USA.

**Keywords:** Bone Biology, Development, Toxicology

**The authors reply:** As representatives for our entire team, we thank Vinayachandran and Balasubramanian (1) for their insightful comments on our recent study investigating the role of male alcohol use in driving alcohol-related craniofacial abnormalities and growth defects (2). In their letter, the authors inquired about developmental abnormalities in the palate teeth. In mice, bone and teeth form during late embryogenesis (E15.5–E19) (3). Therefore, the E16.5 fetal samples we examined did not contain sufficient calcified bone or mineralized teeth to reliably quantify the requested orodental features using available methods of visualization and expertise. However, as we were intrigued by their question and have generated adult offspring as part of a behavioral study, we used dual x-ray absorptiometry (DEXA) scanning to measure basic orodental features, as described previously (4).

We imaged the skulls of 20-week-old male offspring derived from control parings and those after maternal alcohol exposure (MatExp), paternal alcohol exposure (PatExp), and dual parental alcohol exposure (DualExp) (Figure 1, A and B) and measured the spacing and size of the teeth. Consistent with our analysis of E16.5 samples, demonstrating that alcohol-induced changes predominantly impact the lower regions of the face (2), we identified alterations in the inferior but not the superior diastema in PatExp and DualExp offspring (Figure 1, C and D). Furthermore, we found increases in superior and inferior incisor lengths in PatExp offspring and in the width of the inferior molars in the DualExp treatment group (Figure 1, E–G). In addition, we identified decreased distance between the superior molars and incisor tip of adult offspring derived from the MatExp treatment (Figure 1H). Although we acknowledge that these preliminary experiments provide a crude measure of a complex system, they suggest developmental anomalies in the size and spacing of the teeth, which Vinayachandran and Balasubramanian correctly assert are recognized symptoms of fetal alcohol syndrome (FAS) in humans.

These preliminary data reinforce our assertion that FAS craniofacial phenotypes can emerge from paternal alcohol exposures alone and that the exclusive focus on maternal exposures as a diagnostic criterion of FAS may be flawed. Moreover, they suggest that the craniofacial defects described in our E16.5 fetal samples may persist through development into adulthood, emphasizing that FAS is not merely a pediatric disorder but persists across the life course (5). In the future, we will use micro-CT imaging to conduct a more thorough investigation of tooth developmental anomalies, including alterations in jaw alignment and variations in the size of individual teeth. We thank the authors for their enthusiasm and insight.

## Figures and Tables

**Figure 1:**
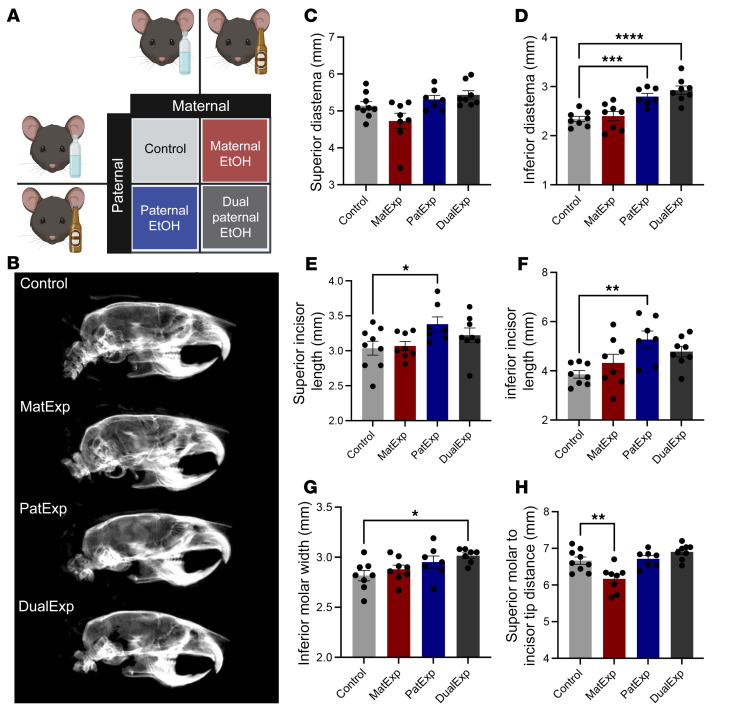
Maternal, paternal, and dual parental alcohol exposures induce changes in adult offspring tooth size and spacing. (**A**) As described previously (2), we used a 2 × 2 factorial design to contrast the offspring of maternal alcohol exposures (MatExp), paternal alcohol exposures (PatExp), and dual parental alcohol exposures (DualExp). (**B**) We used DEXA scanning (Faxitron Pro x-ray cabinet, Hologic Inc.) to image the skulls of 20-week-old adult male offspring (representative scans displayed) and ImageJ (NIH) to determine the size and spacing of the teeth. (**C**–**H**) We used 1-way ANOVA followed by Tukey’s multiple comparisons test to contrast changes in (**C**) the superior diastema, (**D**) the inferior diastema, (**E**) superior incisor length, (**F**) inferior incisor length, (**G**) width of the inferior molars, and (**H**) the distance between the superior incisor tip and molars (*n* = 7–9). Data represent mean ± SEM. *P < 0.05, **P < 0.01, ***P < 0.001, ****P < 0.0001.

## Version 1 10/02/2023

Electronic publication
